# GBF1 and Arf1 interact with Miro and regulate mitochondrial positioning within cells

**DOI:** 10.1038/s41598-018-35190-0

**Published:** 2018-11-20

**Authors:** Laurence Walch, Emilie Pellier, Weihua Leng, Goran Lakisic, Alexis Gautreau, Vincent Contremoulins, Jean-Marc Verbavatz, Catherine L. Jackson

**Affiliations:** 1 Institut Jacques Monod, UMR7592 CNRS Université Paris-Diderot, Sorbonne Paris Cité, Paris, France; 2 Max Planck Institute of Molecular Cell Biology and Genetics, Dresden, Germany; 3 CNRS UMR7654, Ecole Polytechnique, Palaiseau, France

## Abstract

The spatial organization of cells depends on coordination between cytoskeletal systems and intracellular organelles. The Arf1 small G protein and its activator GBF1 are important regulators of Golgi organization, maintaining its morphology and function. Here we show that GBF1 and its substrate Arf1 regulate the spatial organization of mitochondria in a microtubule-dependent manner. Miro is a mitochondrial membrane protein that interacts through adaptors with microtubule motor proteins such as cytoplasmic dynein, the major microtubule minus end directed motor. We demonstrate a physical interaction between GBF1 and Miro, and also between the active GTP-bound form of Arf1 and Miro. Inhibition of GBF1, inhibition of Arf1 activation, or overexpression of Miro, caused a collapse of the mitochondrial network towards the centrosome. The change in mitochondrial morphology upon GBF1 inhibition was due to a two-fold increase in the time engaged in retrograde movement compared to control conditions. Electron tomography revealed that GBF1 inhibition also resulted in larger mitochondria with more complex morphology. Miro silencing or drug inhibition of cytoplasmic dynein activity blocked the GBF1-dependent repositioning of mitochondria. Our results show that blocking GBF1 function promotes dynein- and Miro-dependent retrograde mitochondrial transport along microtubules towards the microtubule-organizing center, where they form an interconnected network.

## Introduction

The membrane-bound organelles of eukaryotic cells are highly dynamic structures, constantly changing their organization and morphology in response to cellular needs. For example, mitochondria can exist either as a large interconnected network or as a collection of individual globular structures^1,2^, and the Golgi apparatus can vary from a large, centrosome-proximal stack of saccules such as that found in many mammalian cells^3,4^, to the dispersed collection of tubular network structures found in yeast^5,6^. Dramatic changes occur during mitosis, when the Golgi apparatus disperses^7,8^, and mitochondria move along microtubules from the cell periphery to the division plane, and then back^1,2,9^. During terminal differentiation, when cells exit the cell division cycle and acquire specialized functions, the positioning and morphology of both mitochondria and the Golgi also change. In particular, the functions of highly polarized cells such as neurons, pancreatic acinar cells and astrocytes depend on the correct spatial distribution of these organelles^1,10–13^.

Small G proteins of the Arf family regulate many aspects of membrane dynamics in cells, including Golgi structure and function^14^ and, as shown recently, mitochondrial morphology and function^15^. Arf proteins switch between inactive GDP-bound and active GTP-bound forms. Arf proteins are tightly membrane-bound in their active form, and recruit a number of proteins, called effectors, to the membrane domains on which they are activated. Guanine nucleotide exchange factors (GEFs) catalyze Arf activation, promoting release of GDP and binding of GTP to the Arf protein through the action of their catalytic Sec7 domain^16,17^. Two subfamilies of Arf GEFs, Gea/GBF1 and Sec7/BIG, carry out essential functions in eukaryotic cells^18,19^. Arf GEFs and Arf small G proteins play important roles in both cell division and in the specialized functions of differentiated cells^14^.

The microtubule cytoskeleton plays a key role in the spatial organization of many organelles, including the endoplasmic reticulum (ER), mitochondria and the Golgi apparatus. Organelle positioning depends on microtubule motors that bind membrane compartments through adaptor proteins and move them towards one or the other end of a microtubule. Cytoplasmic dynein is the major microtubule minus end directed motor, and is part of a very large multimeric complex. Kinesin motors generally move organelles in the opposite direction, towards microtubule plus ends, and also use adaptors to interact with membranes. Miro1 and Miro2 (which we will collectively refer to as Miro) are highly similar transmembrane-domain mitochondrial proteins that bind to adaptor complexes that link either dynein or kinesin motors to the mitochondrial membrane^20–22^. Miro proteins were first identified in mammalian cells as atypical Rho-like GTPases localized to the mitochondrial outer membrane^23,24^. Recently, Lee and coworkers have shown that Miro phosphorylation regulates mitochondrial functions at ER-mitochondria membrane contact sites^25^. An evolutionarily conserved role for Gea/GBF1 and Arf1 in mitochondrial dynamics has been demonstrated recently, which in yeast is mediated by a genetic interaction between Gea1/Gea2 and Gem1, the yeast orthologue of Miro^15^. Whether GBF1 in higher eukaryotes interacts with Miro proteins to mediate the effects of GBF1 on mitochondrial morphology has not been addressed.

In the present study, the involvement of GBF1 and Arf1 in the regulation of mitochondrial network organization in human cells was investigated. Our results show that upon inhibition of either GBF1 or Arf1 function, mitochondria are relocated to a juxta-nuclear region, proximal to the centrosome, where they form a complex network. This condensation of mitochondria towards the microtubule organizing center (MTOC) is dependent on Miro and dynein. We identify Miro as an interaction partner of GBF1, and show that Miro interacts specifically with the active form of Arf1 in co-immunoprecipitation assays. Our data support the conclusion that GBF1 activation of Arf1 blocks Miro-dependent retrograde mitochondrial transport along microtubules towards the MTOC. These results suggest that GBF1 and Arf1 are involved in the regulation of morphology and positioning of mitochondria within cells.

## Results

### GBF1 inhibitors alter mitochondrial network positioning

**Figure 1:**
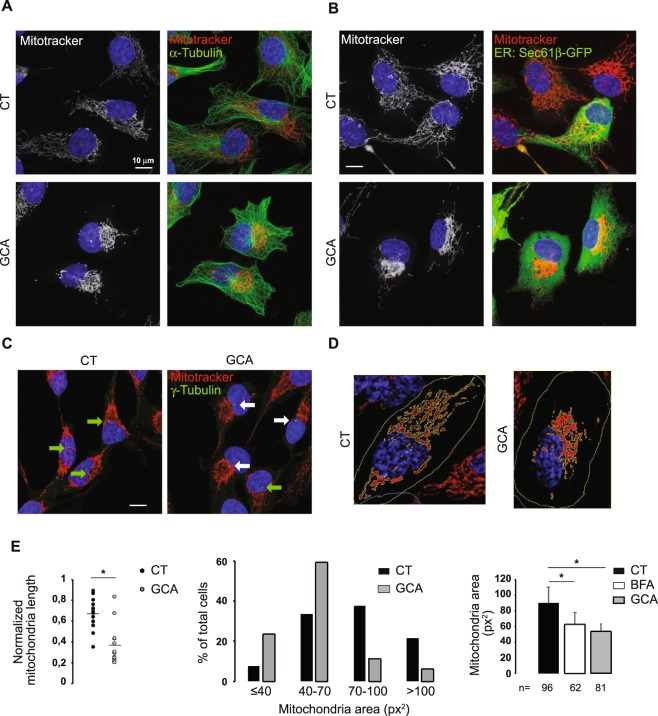
The inhibition of GBF1 induces the condensation of the mitochondrial network in the pericentriolar region without affecting cell shape. RPE1 cells were incubated with either GCA (10 μM) or DMSO alone (CT) for 1h30, were loaded with MitoTracker Orange for an additional 30 min prior to fixation and imaged using confocal microscopy. Images are maximum intensity Z-projections. All bars = 10 µm. Nuclei appear in blue (DAPI staining) and mitochondrial network in white or red. (**A**) Cells were stained for α-tubulin after fixation (green signal). (**B**) RPE1 cells were transfected with the ER marker Sec61β-GFP. (**C**) RPE1 cells were stained for γ−tubulin after fixation (green signal). In cells treated with GCA, the mitochondrial network is frequently condensed in the juxta-nuclear region next to the centrosome (white arrows) but not in control cells (green arrows). (**D**) The mitochondria area was segmented using ImageJ software. The mitochondrial network was converted into masks after the cells were manually contoured. (**E**) Quantification of image analysis. Left: the length of the mitochondrial network in close vicinity of the nucleus was quantified from confocal images using ImageJ (see **D**) in thirteen cells for each cell treatment and normalized by the nucleus perimeter. Bars represent the mean values (0.66 ± 0.153 and 0.37 ± 0.192 for CT and GCA treated cells, respectively). Right: the mitochondria surface area was quantified in 96 (CT) or 81 cells (GCA). Graph shows the distribution of the mitochondria area depending on the cell treatment. Lower: Likewise, the mitochondria surface area was quantified in 62 to 96 cells depending on the cell treatment. Results are the mean ± S.D. of three independent experiments, *p ≤ 0.05 *versus* control cells.

The drug brefeldin A (BFA) has been instrumental in the study of dynamic mechanisms underlying Golgi structure and function^26^. BFA blocks activation of the small G protein Arf1, which results in the disassembly of the Golgi. The precise mechanism of action of the drug has been elucidated^27^. BFA has a high level of specificity, binding only to the catalytic domains of Gea/GBF and Sec7/BIG Arf GEFs in complex with Arf1-GDP, and prevents the activation of Arf1 by one of these GEFs^27,28^. More recently, an even more specific inhibitor was identified, golgicide A (GCA), which binds only to the Sec7 domain of GBF1, and not to the catalytic domains of Sec7/BIG proteins^29^. To establish whether GBF1 may regulate mitochondrial positioning or morphology in mammalian cells, we treated RPE1 cells with BFA or GCA for 2 hours at concentrations known to disrupt the Golgi apparatus^29,30^, then mitochondria were visualized using a cell-permeant MitoTracker. Both treatments induced a significant condensation of the mitochondrial network into the juxta-nuclear region (Fig. 1). This condensation of mitochondria towards the cell center was rapid, as observed in live cells treated with BFA for 30 minutes (Supplementary Movie S1). In contrast, we did not detect alterations in microtubule or ER morphologies, suggesting that cell geometry was not affected (Fig. 1A,B). Co-staining of the mitochondrial network with γ-tubulin revealed that GCA induces the condensation of mitochondria near the centrosome (the MTOC of mammalian cells) (Fig. 1C). The condensation of mitochondria in response to BFA or GCA treatment was more dramatic in a fraction of the cells. In Fig. 1C, white arrows show cells with a condensed mitochondrial network, and green arrows show cells considered normal. This effect was quantified visually in 65 to 98 cells depending on the treatment, and a significant difference was found between control and drug-treated cells (control (CT): 11 ± 4% of cells, BFA: 38 ± 7% and GCA: 26 ± 3%, *p* ≤ 0.05 versus CT). However, drug effects were reflected more objectively by a reduction of the length of the mitochondrial network surrounding the nucleus or the mitochondria surface area, measured using ImageJ macros (Fig. 1D). Despite the cellular variability of the phenotype, quantifications using both methods confirmed a significant condensation of mitochondria after BFA or GCA treatment (Fig. 1E). The latter method (mitochondria surface area on maximum intensity Z projections) was chosen for quantification of drug effects in the following sections.

### GBF1 inhibition enhances retrograde movement of mitochondria

**Figure 2:**
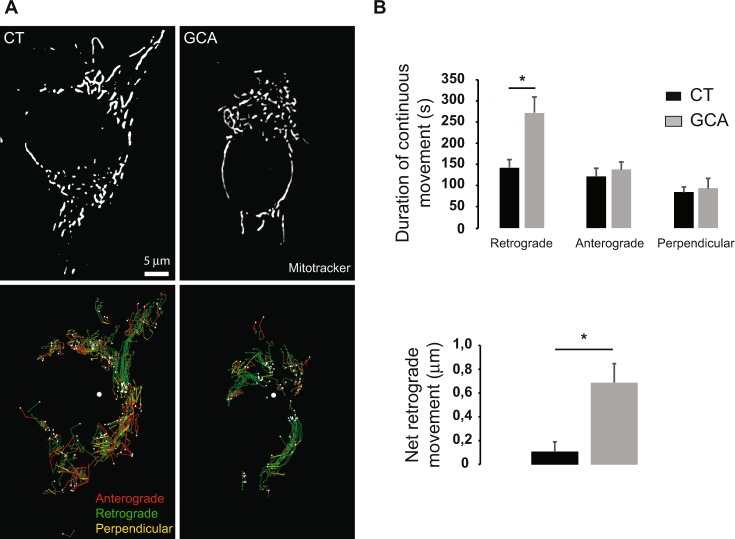
The inhibition of GBF1 increases the net retrograde movement of mitochondria by increasing the duration of retrograde displacements without affecting other movements. RPE1 cells were incubated with MitoTracker Orange for 30 min followed by either GCA (10 μM) or DMSO alone (CT) prior to live-cell imaging for 30 min by spinning disk microscopy. (**A**) The mitochondrial network was segmented over time using ImageJ (Top) and patches of network were tracked over the duration of movies by cross correlation (Bottom, see methods). Retrograde tracking segments, towards the center of mass of the final network (large white dot), are shown in green, anterograde traces, in the opposite direction, in red, and movements within 10° of the perpenticular of antero-retrograde axis in yellow. Small white dots show the final location of patches, and a small green dot indicates the starting point for each patch. (**B**) Top. Mitochondrial patches exhibited stretches of movement along, or perpendicular to the antero-retrograde axis. The duration of continuous retrograde, anterograde or perpendicular movements of mitochondria network patches was quantified in control and GCA-treated cells. Bottom. The net retrograde movement is the difference between average retrograde and anterograde displacements. Results are mean ± S.E.M. (n = 4 cells, 308 traces in controls, 194 traces in GCA-treated cells *p < 0.02).

To determine whether mitochondrial condensation upon GBF1 inhibition was the result of enhanced retrograde trafficking to the cell center, or decreased anterograde transport to the cell periphery, movements of the mitochondrial network after GCA treatment were measured and quantified from live cell imaging (Supplementary Movies S2 and S3). Because mitochondria form a network, patches of the network were identified and tracked in control and GCA-treated cells for 30 min (Fig. 2A). The movement of mitochondria was not monotonous in either condition. It consisted of alternative displacements in different directions. Mitochondria displayed retrograde movements (Fig. 2A green traces), towards a central position (large white spot), anterograde movements (Fig. 2A red traces), and movements perpendicular to the antero-retrograde axis (Fig. 2A yellow traces), until their final location (small white spots). Consistent with the clustering of mitochondria, retrograde movements were dominant in GCA-treated cells (Fig. 2A, right panel), but not in control cells (Fig. 2A, left panel). These movements were quantified (Fig. 2B). Quite strikingly, in control cells, the duration of retrograde and anterograde movements of mitochondrial network patches was extremely similar, with no statistically significant difference observed (Fig. 2B top). As a result, the net movement was close to zero (Fig. 2B bottom panel). In GCA-treated cells, the duration of retrograde displacements was increased by two fold, whereas anterograde or perpendicular movements were identical to control cells. This resulted in a significant net retrograde movement (Fig. 2B, bottom). In a given condition (control or GCA-treated), the speed of movements was the same in any direction, slightly, but not significantly, slower in GCA-trated cells (~0.31 µm/s) than in control cells (~0.43 µm/s). Speed was therefore without effect on the net movement. Our results strongly support the hypothesis that GBF1 inhibition markedly enhances the duration of retrograde movements of mitochondria, without affecting other movements or speed.

### Mitochondrial condensation caused by mutant Arf1 and RNAi against GBF1

**Figure 3:**
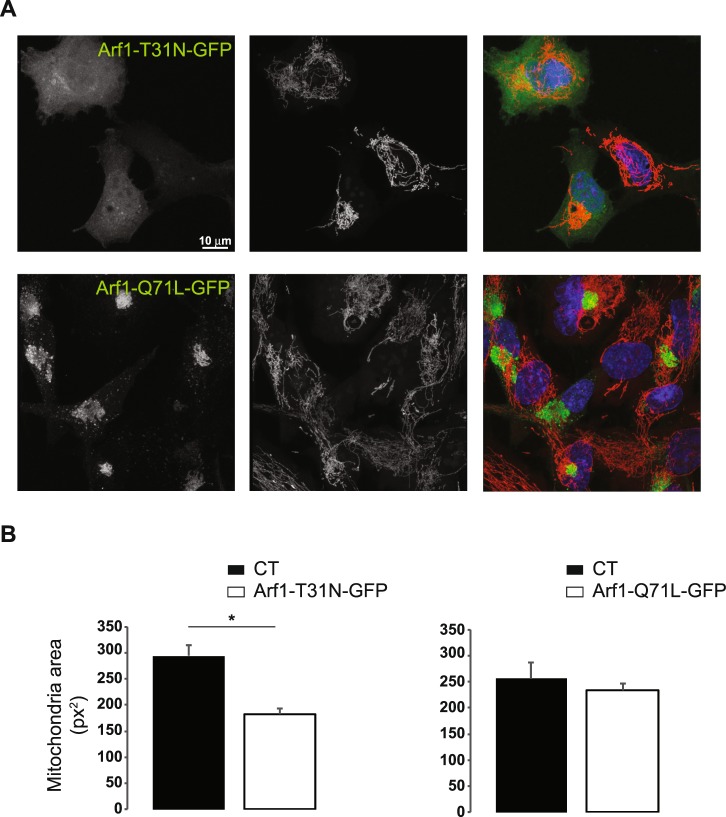
Arf1 deficient mutants induce the condensation of the mitochondrial network. (**A**) RPE1 cells transfected with either the GTP-binding defective (Arf1-T31N-GFP) or the GTP-locked mutant of Arf1 (Arf1-Q71L-GFP) tagged at their C-terminus with GFP were loaded with MitoTracker Orange for 30 min and fixed. Cells were imaged using confocal microscopy. Images are maximum intensity Z-projections. Nuclei appear in blue. (**B**) Mitochondria area was quantified in 12 to 37 cells, depending on the condition, from confocal images using ImageJ software. Results presented are the mean ± S.D. of two independent experiments, *p ≤ 0.05 *versus* control cells. Control cells are cells which did not express the Arf mutant construct.

In order to determine if Arf1 is involved in the condensation of mitochondria upon GBF1 inhibition, RPE1 cells were transfected with the dominant negative mutant of Arf1, Arf1-T31N-GFP, which traps GBF1 in an inactive complex, and disrupts Golgi structure^31^. One day post-transfection, Arf1-T31N-GFP expressing cells exhibited mitochondrial clustering in a juxta-nuclear region (Fig. 3A top panels, B left panel). In contrast, transfection of the constitutively active form of Arf1, Arf1-Q71L-GFP, which causes an enlargement of the Golgi network, had no observable effect on mitochondrial network positioning or morphology (Fig. 3A lower panels, B right panel). The effect of the inactive Arf1 mutant could either be due to GBF1 cytosolic trapping by Arf1-T31N-GFP, or to GBF1 activation of Arf1. Altogether, these results indicate that GBF1 activity is required to maintain the distribution of the mitochondrial network throughout the cell.

Lowering GBF1 expression in RPE1 cells by transfection with GBF1 siRNA duplexes also caused the condensation of the mitochondria into a juxta-nuclear area, around the remaining Golgi structure, where low levels of GBF1 staining were still visible (Supplementary Fig. S1). However, in this situation, the morphology of mitochondria appeared to be somewhat different than that observed in BFA- or GCA-treated cells. In approximately 80% of GBF1 siRNA-treated cells (Supplementary Fig. S1), mitochondria formed condensed structures around the Golgi remnant, but these condensed structures were more dispersed than in drug-treated cells.

**Figure 4:**
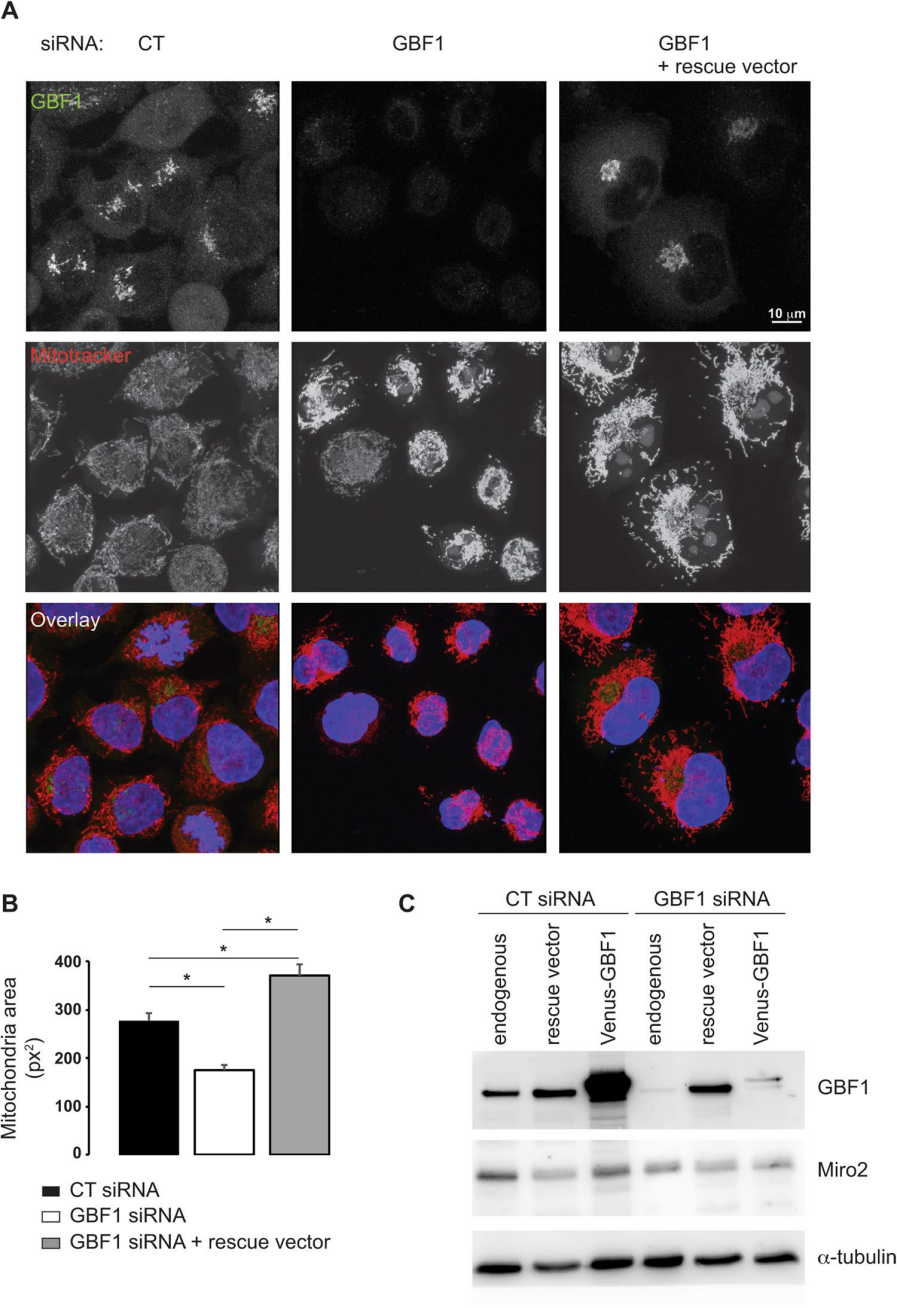
GBF1 silencing causes condensation of the mitochondrial network in HeLa cells. (**A**) HeLa cells were transfected with GBF1, or irrelevant siRNA duplexes (CT), and two days post-transfection, cells were transfected with the hamster GBF1 construct that is resistant to human GBF1 siRNA duplexes (GBF1 + rescue vector). The next day, cells were loaded with MitoTracker Orange for 30 min, fixed and stained for GBF1 (green signal). Cells were imaged using confocal microscopy. Images are maximum intensity Z-projections. Nuclei appear in blue. (**B**) Mitochondria area was quantified in 25 to 46 cells depending on the condition from confocal images using ImageJ software. Results presented are the mean ± S.D. of two independent experiments, *p ≤ 0.05 *versus* control cells. (**C**) In order to ascertain that the hamster GBF1 is resistant to the human GBF1 siRNA duplexes, HeLa cells were transfected with either GBF1 or irrelevant siRNA duplexes. Two days post-transfection, cells were transfected with either the hamster GBF1 or the Human Venus-GBF1 construct. The next day, cells were lysed, endogenous and overexpressed GBF1 levels were analyzed by Western blotting using GBF1 antibodies. The expression level of endogenous Miro2 and of the loading control α-tubulin were also monitored. 20 μg of protein were loaded per lane.

To verify that the mitochondrial phenotype observed upon GBF1 siRNA treatment was not due to off-target effects, we performed a rescue experiment using a heterologous GBF1 cDNA (from hamster). Unfortunately, the double transfection was toxic to RPE1 cells. Hence we turned to HeLa cells. First, we verified that the phenotype of GCA-treated HeLa cells was indistinguishable from that of GCA-treated RPE1 cells (Supplementary Fig. S2). Second, HeLa cells treated with GBF1 siRNA had a condensed mitochondrial phenotype (Fig. 4A), and quantifications indicated a decrease of mitochondrial area in siRNA-treated compared to control cells (Fig. 4B). HeLa cells remained viable upon double transfection with GBF1 siRNA and the rescue plasmid from hamster. We demonstrated that hamster GBF1 expression was resistant to our GBF1 siRNA treatment (Fig. 4C), and that hamster GBF1 cDNA restored normal mitochondrial morphology (Fig. 4A,B). Together, these results show that GBF1 inhibition by either drug or GBF1 siRNA treatment causes a condensation of the mitochondrial network in cells.

In HeLa cells, as in RPE1 cells, the mitochondrial network is less condensed upon GBF1 siRNA treatment compared to drug treatments. This difference may be due to different modes of GBF1 inhibition, i.e. rapid stabilization of an Arf1-GDP-GBF1 complex for BFA and GCA, in contrast to long-term, but incomplete, reduction in the overall level of GBF1 (Figs 4C and S1), and the presence of Golgi remnants, after siRNA treatment (Supplementary Fig. S2). Because mitochondrial condensation is observed in siRNA-treated RPE1 and HeLa cells, yet many Golgi elements remain at least partially intact (Supplementary Fig. S2), we can conclude that the mitochondrial phenotype is not simply a secondary effect of Golgi disassembly.

### Identification of Miro as a GBF1 binding partner

**Figure 5:**
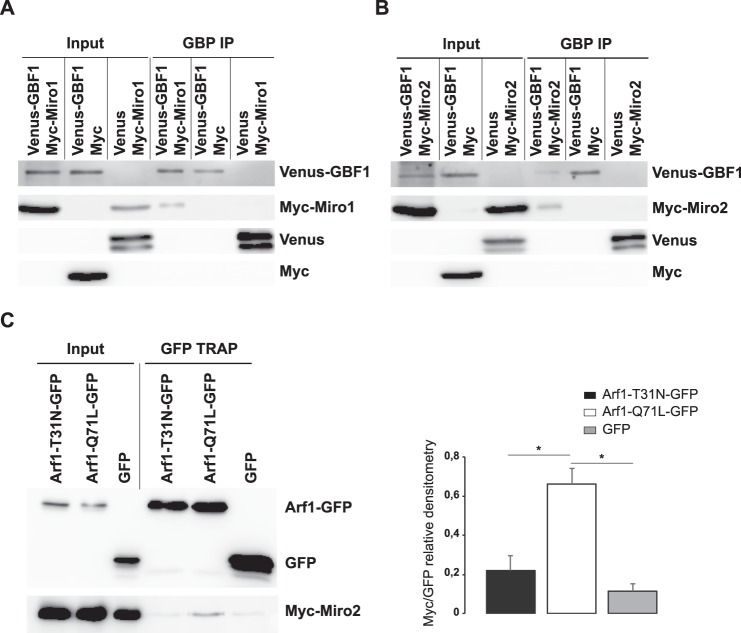
Miro GTPases interact physically with GBF1. (**A**,**B**) RPE1 cells were cotransfected with Venus or Venus-tagged GBF1 and either Myc or Myc-Miro1 (**A**) or Myc-Miro2 (**B**). Immunoprecipitations were carried out with GBP-beads. Cells lysates (Input) and immunoprecipitated proteins were analyzed by Western blotting using anti-GFP and anti-Myc antibodies. The input lanes represent 15% of lysate used in immunoprecipitation reactions. (**C**) RPE1 cells were cotransfected with Arf1-T31N-GFP, Arf1-Q71L-GFP or GFP and Myc-Miro2. GFP-tagged proteins were immunoprecipitated with magnetic GFP-Trap^®^_A. Cells lysates (Input) and immunoprecipitated proteins were analyzed by Western blotting using GFP- and Myc-antibodies. The input lanes represent 10% of lysate used in immunoprecipitation reactions. One representative experiment of three independent experiments is shown. Bands were quantified by a densitometric analysis performed using Image J software. Results are the mean of the ratio between the Myc and the GFP band values ± S.D. of the three independent experiments. *P ≤ 0.05 *versus* control cells.

Using a tandem affinity purification/mass spectrometry strategy in HEK293 cells, we have identified Miro2 as one putative GBF1 binding partner (Supplementary Fig. S3). To verify the interaction of GBF1 with Miro1 and Miro2 in RPE1 cells, we performed pull-down experiments in cells expressing Venus-GBF1 and either myc-Miro1 (Fig. 5A) or myc-Miro2 (Fig. 5B). The results show that a small fraction of both Myc-Miro1 and Myc-Miro2 co-immunoprecipitate with Venus-GBF1 (Figs 5A,B and S3), suggesting that GBF1 and Miro are physically associated. The relatively small level of interaction is consistent with GBF1 localizing only transiently to membranes, cycling rapidly on and off the cytosolic surfaces of organelles, as has been demonstrated for the Golgi^30^. Because Miro2 appeared to be the major Miro isoform expressed in RPE1 cells (Supplementary Fig. S4), we will focus exclusively on Miro2 in the following experiments. In order to evaluate if Arf1 also interacts with Miro2, cells were co-transfected with Myc-Miro2 and either inactive Arf1-T31N-GFP or constitutively active Arf1-Q71L-GFP, and the Arf1 mutants were immunoprecipitated using GFP Trap. A small pool of Myc-Miro2 was immunoprecipitated with Arf1-Q71L-GFP (Fig. 5C), but not with Arf1-T31N-GFP (Fig. 5C), indicating a selective interaction between Miro2 and the active form of Arf1. These results show that Miro2 is either an effector of Arf1, or is part of an Arf1 effector complex, that interacts with GBF1 at the mitochondrial membrane.

### Miro2 and dynein activity are required for the BFA/GCA effect on the mitochondrial network

**Figure 6:**
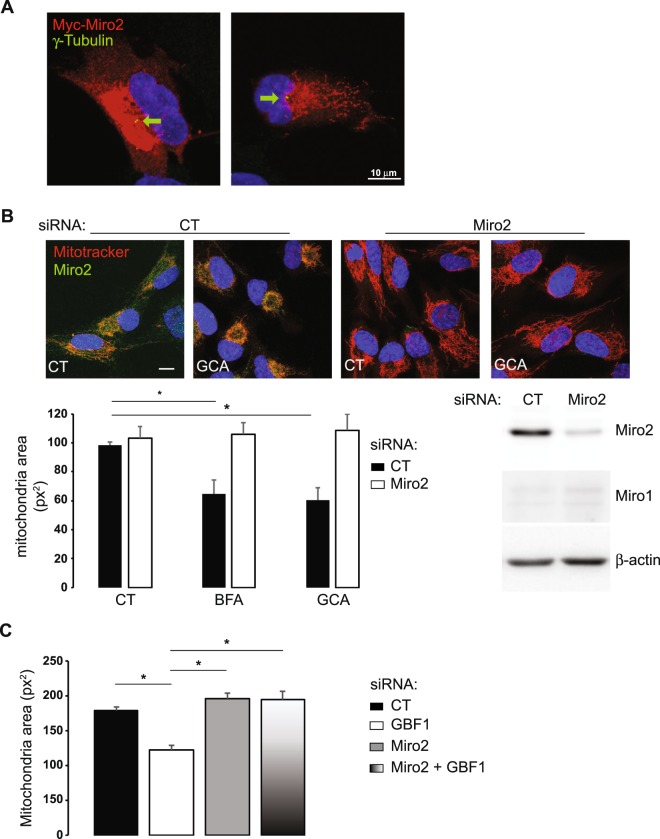
Miro2 is required for GBF1-dependent mitochondrial positioning in RPE1 and HeLa cells. (**A**) RPE1 cells were transfected with Myc-Miro2, fixed and stained for Myc (red signal) and γ−tubulin (green signal). Images are maximum intensity Z-projections. Nuclei appear in blue. The green arrow indicates the centrosome. (**B**) RPE1 cells were transfected with either Miro2 siRNA duplexes or (CT) irrelevant siRNA duplexes. Two days post-transfection, cells were incubated with either BFA (10 μg/ml), GCA (10 μM) or DMSO alone (CT) for 1h30, loaded with MitoTracker Orange for an additional 30 min and fixed. Upper panel shows cells stained for Miro2 (green signal). Nuclei appear in blue (DAPI staining) and mitochondrial network in red. Images are maximum intensity Z-projections. Lower left panel shows mitochondria area quantified using ImageJ software. Results are the mean ± S.D. of three independent experiments in 78 to 102 cells depending on the cell treatment, *P ≤ 0.05 *versus* control cells. Lower right panel shows Western blot analysis performed two days post-transfection to monitor the expression level of Miro and of the loading control β-actin in cells treated with siRNA duplexes. (**C**) HeLa cells were transfected with GBF1 or Miro2 alone, GBF1 and Miro2 or (CT) irrelevant siRNA duplexes. Three days post-transfection, cells were loaded with MitoTracker Orange, fixed and stained for GBF1 or Miro2. Mitochondria area was quantified in 51 to 83 cells depending on the condition from confocal images (see Supplementary Fig. S5) using ImageJ software. Results presented are the mean ± S.D. of two independent experiments, *p ≤ 0.05 *versus* control cells.

It is well established, particularly in neuronal systems, that Miro1 and Miro2 regulate mitochondrial trafficking and positioning along microtubules^12,20,32^. To assess if Miro2 may contribute to GBF1-dependent mitochondrial network remodeling, we first expressed Myc-Miro2 in RPE1 cells. In these Myc-Miro2 transfected cells, mitochondria were condensed near the MTOC, as visualized by γ-tubulin staining (Fig. 6A), which has been reported previously^24^. This result suggests that Miro2 overexpression alone results in the same phenotype as BFA/GCA treatment (Fig. 6A). More importantly, lowering Miro2 expression by transfection with specific siRNA duplexes abolished BFA- and GCA- induced mitochondrial condensation to the pericentrosomal region (Fig. 6B). Miro2 siRNA duplexes had no effect on mitochondrial network positioning in untreated cells (Figs 6C and S5) or on Miro1 expression (Fig. 6B). Finally, transfection of Miro2 siRNA duplexes together with GBF1 siRNA prevented the effect of GBF1 siRNA treatment on the spatial organization of the mitochondrial network (Figs 6C and S5). Together, our results suggest that Miro interacts physically with GBF1 and mediates the effects of GBF1 on intracellular positioning of mitochondria.

**Figure 7:**
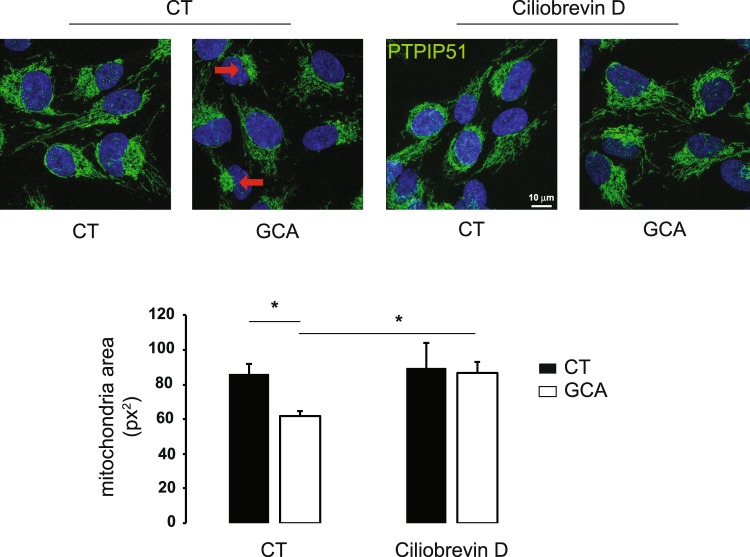
The inhibition of cytoplasmic dynein motor activity reduces GBF1 inhibitor GCA-mediated mitochondrial clustering. RPE1 cells were incubated either with cytoplasmic dynein inhibitor, Ciliobrevin D (50 μM), or DMSO alone (CT) for 15 min and for an additional 2 h with either BFA (10 μg/ml), GCA (10 μM) or DMSO alone (CT). Cells were fixed and stained for the mitochondrial marker, PTPIP51. Cells were imaged using confocal microscopy. Mitochondria area was quantified using ImageJ. Results are the mean ± S.D. of three independent experiments in 77 to 96 cells depending on the cell treatment, *P ≤ 0.05 *versus* control cells.

Miro-dependent trafficking of mitochondria is dependent on the interaction between Miro and the microtubule motor proteins kinesin and dynein^20,21^. To assess if the retrograde transport of the mitochondrial network upon BFA/GCA treatment in RPE1 cells is dependent on microtubule minus end directed dynein transport, we studied the effect of BFA and GCA in cells pretreated with ciliobrevin D, a cytoplasmic dynein inhibitor. As shown in Fig. 7, ciliobrevin D pretreatment abolished the GCA-induced condensation of mitochondria towards the centrosomal region, whereas ciliobrevin D alone had no detectable effect on the mitochondrial network. Similar results were obtained in cells treated with BFA (not shown). Together with the mitochondrial tracking experiments (Fig. 2), these results support the conclusion that mitochondrial retrograde movement upon GBF1 inhibition is mediated by dynein along microtubules.

### Mitochondrial morphology is altered by GBF1 inhibition

**Figure 8:**
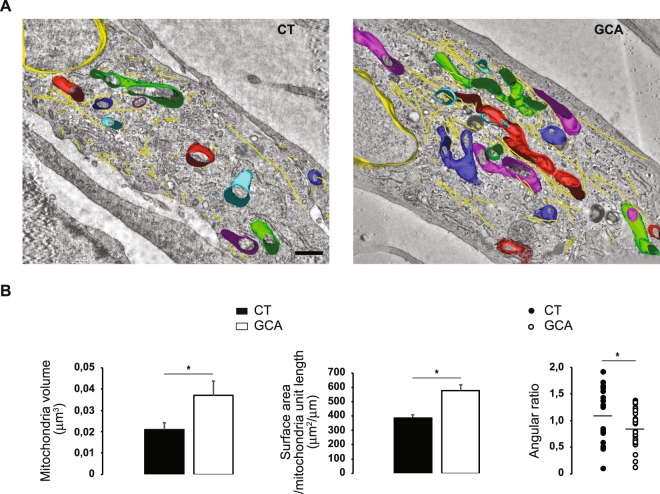
The inhibition of GBF1 induces a condensation and a change of morphology of mitochondria. RPE1 cells were incubated with either GCA (10 μM) or DMSO alone (control) for 1h30 and juxta-nuclear regions were imaged by electron tomography, followed by segmentation of mitochondria (blue), microtubules (red) and the nucleus (pink). (**A**,**C**): control cells. (**B**,**D**): GCA-treated cells. (**A**,**B**): segmentation of longitudinal RPE1 cell sections overlayed on a central tomographic slice, (**C**,**D**): segmentation of cross sections of RPE1 cells. Bar = 0.5 µm.

In order to determine if the relocalization of mitochondria upon GBF1 inhibition also induced a change in mitochondrial morphology, the ultrastructure of mitochondria was studied by electron microscopy in GCA-treated and control RPE1 cells by electron tomography on semi-thin sections. Mitochondria and microtubules were segmented in 3D (Fig. 8). In control cells, mitochondria were relatively circular and dispersed. In GCA-treated cells, mitochondria were consistently condensed in a juxta-nuclear region and exhibited a more complex morphology (Fig. 8A). A quantitative analysis after segmentation of mitochondria and microtubules in tomograms from several cells (4 control cells, 5 GCA-treated cells) showed significant alterations of mitochondrial morphology and spatial organization after GCA treatment (Fig. 8B). In GCA-treated cells, mitochondria were bigger, (75% volume increase, p < 0.05 Fig. 8B left graph) and exhibited a more complex morphology as shown by a 20% increase in the surface area of outer membrane per micron of mitochondrion length (p < 0.05 Fig. 8B, center graph). However, there was no significant difference in the dimension of mitochondria (longest length measured). These results demonstrate an increase in mitochondria connectivity, either through packing of long mitochondria into a smaller volume, or through fusion of smaller mitochondria at the site of clustering. In both control cells and GCA-treated cells, the average orientation of mitochondria was parallel to the average direction of microtubules (not shown), in support of microtubule dependent orientation of mitochondria. However, the angular spread of the mitochondrial long axis with respect to the microtubule network was smaller in GCA treated cells than in control cells (average angular ratio of 0.85 in GCA cells, and 1.10 in control cells, Fig. 8B, right graph, p < 0.05), indicating a better alignment of mitochondria with microtubules in drug-treated cells. ER-mitochondrial contact sites are believed to play a role in mitochondrial fission processes and in their metabolism. Despite the change in mitochondrial position and morphology, we did not find changes in the frequency or the morphology of ER-mitochondria contact sites by electron tomography in GCA-treated RPE1 cells (not shown). These observations suggest that upon inhibition of GBF1, mitochondria are transported along microtubules towards the MTOC, where they form a hyper-connected network.

## Discussion

To date, the best-studied function of GBF1 has been in the secretory pathway, where Arf1 activation by GBF1 regulates retrograde vesicular transport from the cis-Golgi and the ERGIC to the ER. This function of GBF1 is important to maintain Golgi structure and secretory function, and is consistent with the major steady-state localization of GBF1 at the cis-Golgi. Although GBF1 is primarily found at the cis-Golgi, it also localizes to other organelles such as the trans-Golgi and lipid droplets^33,34^.

**Figure 9:**
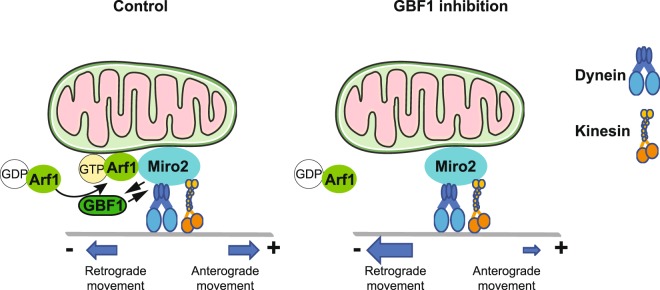
Model of how GBF1, Arf1, Miro2 and dynein may regulate mitochondrial positioning. Under normal conditions (left panel), anterograde and retrograde movements of mitochondria are approximately equivalent. When GBF1 is inhibited (right panel), dynein-mediated retrograde movement is enhanced, leading to a more condensed mitochondrial network near the centrosome at the cell center. See text for additional details.

In yeast and *C*. *elegans*, Arf1 and GBF1/Gea were also shown to be required for mitochondrial morphology, through genetic interactions with Miro/Gem1^15^. Our data reveal that in human cells, a pool of GBF1, and active Arf1-GTP, physically interact with Miro, and we show that inhibition of GBF1 function causes condensation of mitochondria near the MTOC, where they become more connected. Strikingly, inhibition of GBF1 results in a doubling of the time mitochondria engage in retrograde-directed movement, without changing the amount of time spent in movements in other directions. We demonstrate that the movement of mitochondria in the absence of GBF1 function is dependent on the minus end-directed motor dynein and on Miro2 in RPE1 cells. Therefore, our data support the conclusion that GBF1 regulates dynein-mediated retrograde transport of mitochondria along microtubules towards the cell center, with an increase in retrograde movement occurring when GBF1 is inhibited (Fig. 9). We also demonstrate a physical association between the Miro2 dynein adaptor complex and either GBF1 or constitutively active Arf1. However, our results do not demonstrate a direct interaction, or whether this interaction is directly responsible for the movements of mitochondria. In the future, it will be interesting to determine how these physical interactions might be involved in GBF1/Arf1 regulation of retrograde mitochondrial movement. Much is known about Miro regulation of kinesin motors, but recent studies have shown that Miro also interacts with dynein through TRAK/Milton in neurons^35^, and through CenpF in dividing cells^36^.

The yeast Miro orthologue Gem1 is a regulator of endoplasmic reticulum-mitochondria contacts and is also involved in mitochondrial fission^37,38^. There is also evidence that Miro plays a role at ER-mitochondria contact sites in mammalian cells (Kornmann, *et al*., 2011; Lee, *et al*., 2016), and may play a role in mitochondrial fission in Drosophila^39^. We have not observed a change in overall ER distribution, or in ER-mitochondria contacts after mitochondria repositioning induced by GBF1 inhibition. However, we found an increase in mitochondrial volume and network complexity, consistent with increased fusion upon GBF1 inhibition. In any case, the role of Miro and Gem1 at ER-mitochondria membrane contact sites and their regulation by Arf1 and its GBF/Gea GEFs are evolutionarily conserved features, and provide a new perspective to explore the links between mitochondrial positioning and fission/fusion processes mediated by ER-mitochondrial membrane contact.

Although GBF1 itself has not yet been shown to regulate microtubule motor activity at other organelles, active Arf1 has been implicated in dynein-mediated movement of the early Golgi^40^. However, in contrast to our results at mitochondria, Arf1-GTP has been shown to promote the centrosomal localization of the Golgi, through recruitment of the dynein receptor golgin160 to Golgi membranes^40^. Taken together, these results lead to the prediction that inhibition of Arf1 activation would cause opposite movement of mitochondria and Golgi elements along microtubules, with mitochondria becoming more concentrated at the minus ends and the Golgi becoming more dispersed due to net plus end movement. It is interesting to note that during mitosis, when both the mitochondrial network and the Golgi apparatus undergo dramatic structural changes^41^, GBF1 activity is inhibited through phosphorylation by AMPK^42^. Therefore, given that GBF1 has a known role in mitosis^43^, another attractive hypothesis to investigate is whether GBF1 might coordinate the reorganization of the Golgi apparatus and the mitochondrial network during cell division.

## Methods

### Plasmids and primary antibodies

All constructs were confirmed by DNA sequencing. pTKN101 (pVenus-GBF1) has been described previously^30^. pCMS-EGFP-Arf1-T31N (pArf1-T31N-GFP) and pCMS-EGFP-Arf1-Q71L (pArf1-Q71L-GFP) were constructed by insertion of Arf1 mutant cDNAs into pCMS-EGFP (Clontech, Invitrogen) using BamHI and AgeI restriction enzyme sites, placing the mutant Arf proteins under control of the SV40 promoter. pRK5-myc-Miro1 (Plasmid 47888), pRK5-myc-Miro2 (Plasmid 47891) and pAc-GFP-Sec61b (Plasmid 15108) were purchased from Addgene (Cambridge, MA, USA). GFP-3x-Nup160 was a gift from Valérie Doye, Institut Jacques Monod - UMR7592 CNRS Université Paris-Diderot, Paris, France, and has been described^44^. Cricetulus griseus (hamster) GBF1 was a gift of Paul Melançon (University of Alberta, Edmonton, Canada)^45^. The following mouse monoclonal antibodies were used: anti-GBF1 (BD Biosciences, Franklin Lakes, NJ, USA, Cat. No 612116), anti-Miro1 (Sigma-Aldrich, St Louis, MO, USA, Cat. No SAB1407648), anti-α-tubulin (GeneTex, Irvine, CA, USA, Cat. No GTX628802), anti-γ-tubulin (Sigma-Aldrich, Cat. No T6557), anti-c-myc (Biolegend, San Diego, CA, USA, Cat. No 626802) and anti-GFP (Roche Diagnostics, Indianapolis, IN, USA, Cat. No 11814460001). Rabbit polyclonal antibodies against Miro2 (Cat. No HAP012624) and PTPIP51/RMDM3 (Cat. No HPA009975) were purchased from Sigma-Aldrich.

### Drugs

Brefeldin A (BFA), the primary targets of which are the high molecular weight Arf1 GEFs GBF1, BIG1 and BIG2, and the highly specific GBF1 inhibitor, golgicide A (GCA), were obtained from Sigma-Aldrich and used as described previously^15,27,29^. The cytoplasmic dynein inhibitor, ciliobrevin D, was purchased from EMD Millipore (Billerica, MA, USA). Stock solutions were prepared in DMSO.

### Cell culture and transient transfection

Diploid telomerase-immortalized human retinal pigment epithelial hTERT-RPE1 cells (Clontech, Mountain View, CA, USA) were grown in Dulbecco’s modified Eagle medium (DMEM)/Ham F12 medium supplemented with 10% fetal bovine serum, 100U/ml penicillin and 100 μg/ml Streptomycin at 37 °C, in a humidified 5% CO_2_ atmosphere. HeLa cells were cultured in Dulbecco’s modified Eagle’s medium (DMEM) supplemented with 4.5 g/l glucose (Life technologies), 10% fetal bovine serum (FBS, Life technology) and 1% Penicillin and Streptomycin (Life technologies). Cells were transfected with lipofectamine 2000 (using 600- or 400 ng of DNA for a 2 cm^2^ well) lipofectamin RNAimax (RPE1) or oligofectamine (HeLa) (using 80 pmol siRNA for a 10 cm^2^ well). All products mentioned above were obtained from Life Technologies (Carlsbad, CA, USA). On-Target plus Human GBF1 (8729) siRNA-Smart pool and On-Target plus Human RHOT2/Miro2 (89941) siRNA-Smart pool were purchased from GE Healthcare (Little Chalfont, UK). DsiRNA control sequences (NC1 Negative Control Sequence) were obtained from Integrated DNA Technologies (Newark, NJ, USA).

### Immunoprecipitation

Cells cultured in 6-well plates were disrupted with 300 μl of cold lysis buffer (50 mM Tris-HCl (pH 7.5), 100 mM NaCl, 1 mM EDTA, 0.5% NP40) supplemented with a protease inhibitor mix (GE Healthcare). Lysates were preclarified by centrifugation (14,000 g, 4 °C, 15 min). Immunoprecipitations were performed using purified GFP Binding Protein (GBP)^46^ bound covalently to AminoLink resin, according to the manufacturer’s instructions (Thermo Scientific Pierce), after pre-clearing with AminoLink resin alone. 200 μl of cell lysate was incubated with 25 μl of GBP-AminoLink resin for 1h30min at 4 °C under constant stirring. Alternatively, GFP-tagged proteins were immunoprecipitated with magnetic GFP-Trap^®^_A (Chromotek, Planegg, Germany). After extensive washing with cold W100 buffer (50 mM Tris-HCl pH 7.5, 100 mM NaCl, 1 mM EDTA), immunoprecipitates were finally eluted from the beads by boiling for 5 min in Laemmli sample buffer, then were submitted to Western blot analysis.

### Western Blotting

Samples in Laemmli’s buffer were separated by SDS-PAGE. Proteins were then transferred onto nitrocellulose blotting membranes (GE Healthcare). Membranes were blocked with TBS-Tween containing 5% skim milk for 1 h and then incubated 2h30 with primary antibody (anti-GFP, 0.4 μg/ml; anti-c-myc, 1 μg/ml; anti-Miro1, 1 μg/ml and anti-Miro2, 0.8 μg/ml) at 4 °C with stirring. After washing with TBS-tween, membranes were incubated with the suitable secondary antibody coupled to peroxidase (GE Healthcare, Cat. No NA934V, NA931V), and immune complexes were revealed by enhanced chemiluminescence (ECL Select Western Blotting Detection Reagent, GE Healthcare) and visualized using the LAS-3000 Imaging System from Fujifilm (Tokyo, Japan).

### TAP-tag purification and Mass Spectrometry

The GBF1 coding sequence was cloned into pcDNA5/FRT/TO1/His-PC-TEV-Blue, to fuse His_6_ and Protein C (PC) tags (followed by a TEV cleavage site) to the N-terminus of the GBF1 protein. HEK293 cells were transfected with this pcDNA5/FRT/TO1-His_6_-PC-TEV-GBF1 construct to generate a stable HEK293 cell line by directed homologous recombination, as described previously (Derivery *et al*., 2009). His_6_-PC-GBF1 expression was induced with 7 µg/ml of tetracycline for 40 h. Tandem affinity purification was carried out by preparing lysates from the His_6_-PC-GBF1 HEK293 stable cell line in 200 mM NaCl, 1 mM CaCl_2_, 1% Triton X-100, 5% glycerol, 50 mM Na–HEPES, pH 7.4, and incubating first with protein C beads (Roche) then Ni^2+^ Sepharose beads (GE Healthcare), as described previously^47^. After extensive washing, Ni^2+^-Sepharose beads were boiled in SDS loading buffer, bands were cut from the gel, and then analyzed by mass spectrometry. Two subunits of the COPI coat were identified, validating the approach, as we have previously identified the COPI complex as an interacting partner of GBF1^48^.

### Immunocytochemistry and confocal microscopy

The staining of mitochondria was performed in live RPE1 cells grown on coverslips by incubation with the cell-permeant probe MitoTracker Orange CMTMROS (0.1 μM, Life Technologies) for 30 min at 37 °C in DMEM/Ham F12 medium. For immunofluorescence analysis, RPE1 or HeLa cells were washed with PBS and fixed with 4% formaldehyde in PBS for 15 min. The reaction was stopped with 50 mM NH_4_Cl. Cells were blocked and permeabilized for 15 min with a solution of PBS containing 0.5% BSA and 0.1% Triton X-100. Wells were then incubated with the suitable primary antibody against GBF1 (2 μg/ml), Miro2 (4 μg/ml), PTPIP51 (1 μg/ml), α-tubulin (0.1 μg/ml), γ-tubulin (1/200) or c-myc (0,5 μg/ml) for 1 h at room temperature. After several washes in blocking buffer, cells were labeled with the appropriate Alexa Fluor 488 or 555-conjugated secondary antibody (4 μg/ml, Life Technologies, Cat. No A11029, A21429 and A11034). Finally, slides were mounted in Duolink *In Situ* Mounting Medium with DAPI (Sigma-Aldrich), and cells were imaged using a confocal microscope (LSM780, Zeiss). Maximum intensity z-projections of confocal z-stacks were obtained using ImageJ software.

### Mitochondrial network area and length quantification

To measure the surface area of mitochondria on maximum intensity Z-projections, an image processing algorithm was developed in ImageJ to quantify the ratio between the total fluorescence intensity of mitochondria and the mitochondrial area for each cell. All the cells were processed with the same parameters. Total fluorescence intensity of mitochondria was calculated by thresholding the image with the default ImageJ thresholding tool, and the total mitochondrial area contour was determined by thresholding the low pass filtered image. The result was obtained by computing the ratio between the two values. To measure the length of the network, another approach was also used in ImageJ. The mitochondrial area contour was determined as above, and the longest dimension of the network was measured and divided by the perimeter of the nucleus for normalization.

### Mitochondrial network tracking

Live cell imaging of mitochondria was performed on a spinning disk microscope after labeling cells with mitotracker. Cells were treated with DMSO alone or GCA in DMSO and images were recorded at 30 s intervals for 30 min at a resolution of 10 pixels per micron. An image processing algorithm was developed for tracking of the mitochondrial network. For each time point, the average intensity projection of 3 confocal slices was calculated. Cells of interest were outlined manually and drift correction was applied in ImageJ on the whole cell. Then, segmentation of the cell mitochondrial network was performed for each time-point of the movie by background substraction of median-filtered images. An automatic threshold was applied to generate a mask for the network and this mask was applied to the grey level images. Then for each cell, the first image of the movie was cut into a mosaic of 8 × 8 pixels to identify mitochondria-positive ROIs. Each ROI was then expanded to a 64 × 64 pixel patch, corresponding to a small fragment of the mitochondrial network. Each of the patches was tracked across the movie by cross correlation: the new position of the positive patch was identified in the next image and a new reference image, taking into account changes in the mitochondrial network shape, was taken for correlation with the next image. Then, the center of mass of the mitochondrial network at the end of the movie was used as a central reference. Displacements towards this central reference were referred to as “retrograde” and movements opposite to that direction were referred to as “anterograde”. Finally, movements within 10° of the perpendicular to the antero-retrograde axis were named “perpendicular”. The time between changes of direction was measured for each of the patches. The “net retrograde movement” is the difference between the averages of retrograde and anterograde displacements.

### Electron microscopy and tomography

RPE1 cells were grown in 6 well plates, treated with GCA (10 µM) or DMSO alone as a control for 1 h30min. The cells were fixed with 1% glutaraldehyde and 3% formaldehyde in PBS, followed by post fixation in reduced osmium containing potassium ferrocyanide and 1% osmium tetroxide for 1h. The cells were stained pre-embedding with uranyl acetate prior to dehydration in ethanol and embedded in epoxy resin (Durcupan). 300 nm-thick semi-thin sections were cut, counter-stained in lead-citrate and uranyl acetate and imaged by electron tomography (Tecnai F30, 300 kV, FEI, the Netherlands), using a Gatan US-1000 camera and the SerialEM acquisition software^49^. Electron tomograms were reconstructed using the IMOD software^50^. The segmentation of mitochondria was performed manually in IMOD on computer-generated sections, whereas microtubule segmentation was performed in Amira using automated segmentation, followed by manual validation^51^. Quantitative parameters from EM models were extracted using IMOD imodinfo prior to statistical analysis.

### Quantification and statistical analysis

For statistical analysis, values are expressed as mean ± S.D. (mean ± S.E.M. for electron tomography) of at least three independent experiments. The significance of differences between groups was tested using the Student’s *t* test on unpaired samples. A value of *p* ≤ 0.05 was considered significant. Statistical analysis was performed using the Sigmastat software.

## Electronic supplementary material

Supplementary Information

Movie S1.

Movie S2.

Movie S3.

## Data Availability

The data generated and analysed during the current study are available from the corresponding authors on reasonable request.
