# Supplementary material for: GBF1 and Arf1 interact with Miro and regulate mitochondrial positioning within cells
Source: Sci Rep. 2018 Nov 20;8:17121. doi: 10.1038/s41598-018-35190-0 (PMC6244289; doi:10.1038/s41598-018-35190-0)
Supplement: Supplementary file 1 — Supplementary Information [file 41598_2018_35190_MOESM1_ESM.pdf]

GBF1 and Arf1 interact with Miro and regulate mitochondrial positioning within cells

Laurence Walch[1], Emilie Pellier[1], Weihua Leng[2], Goran Lakisic[3], Alexis Gautreau[3], Vincent Contremoulins[1], Jean-Marc Verbavatz[1]*, Catherine L. Jackson[1]*

[1] Institut Jacques Monod, UMR7592 CNRS Université Paris-Diderot, Sorbonne Paris Cité, Paris, France
[2] Max Planck Institute of Molecular Cell Biology and Genetics, Dresden, Germany
[3] CNRS UMR7654, Ecole Polytechnique, Palaiseau, France.

* correspondence:
Jean-Marc Verbavatz jean-marc.verbavatz@ijm.fr
Catherine L. Jackson cathy.jackson@ijm.fr

Supplementary Information:

Supplementary Figure S1. GBF1 silencing affects the mitochondrial network in RPE1 cells.

Supplementary Figure S2. Golgi and mitochondrial morphology in cells treated with GBF1 inhibiting drugs or siRNAs.

Supplementary Figure S3. Co-immunoprecipitation of Miro with GBF1.

Supplementary Figure S4. Miro2 is the predominant Miro isoform in RPE1 cells.

Supplementary Figure S5. Miro2 is required for mitochondrial condensation in HeLa cells knocked down for GBF1.

Supplementary Figure S6. Full-length blots.

Supplementary Movie 1. Effect of BFA on mitochondrial dynamics.

Supplementary Movie 2. Tracking of mitochondrial dynamics in control cells.

Supplementary Movie 3. Tracking of mitochondrial dynamics in GCA-treated cells.

Figure S1. GBF1 silencing affects the mitochondrial network in RPE1 cells.
Cells were transfected with GBF1, or irrelevant siRNA duplexes (CT). (A) Three days post-transfection, cells were loaded with MitoTracker Orange, fixed and stained for GBF1 (green signal). Images are maximum intensity Z-projections. (B) The number of cells with a mitochondrial network appearing like interconnected globular structures was counted in three independent experiments (right panel). The quantification was performed in a total of 99 (CT) to 105 (siRNA GBF1) cells (C) Three days post-transfection, Western blot analysis was performed to monitor the expression level of GBF1 and of the loading control β-actin in cells treated with siRNA duplexes.

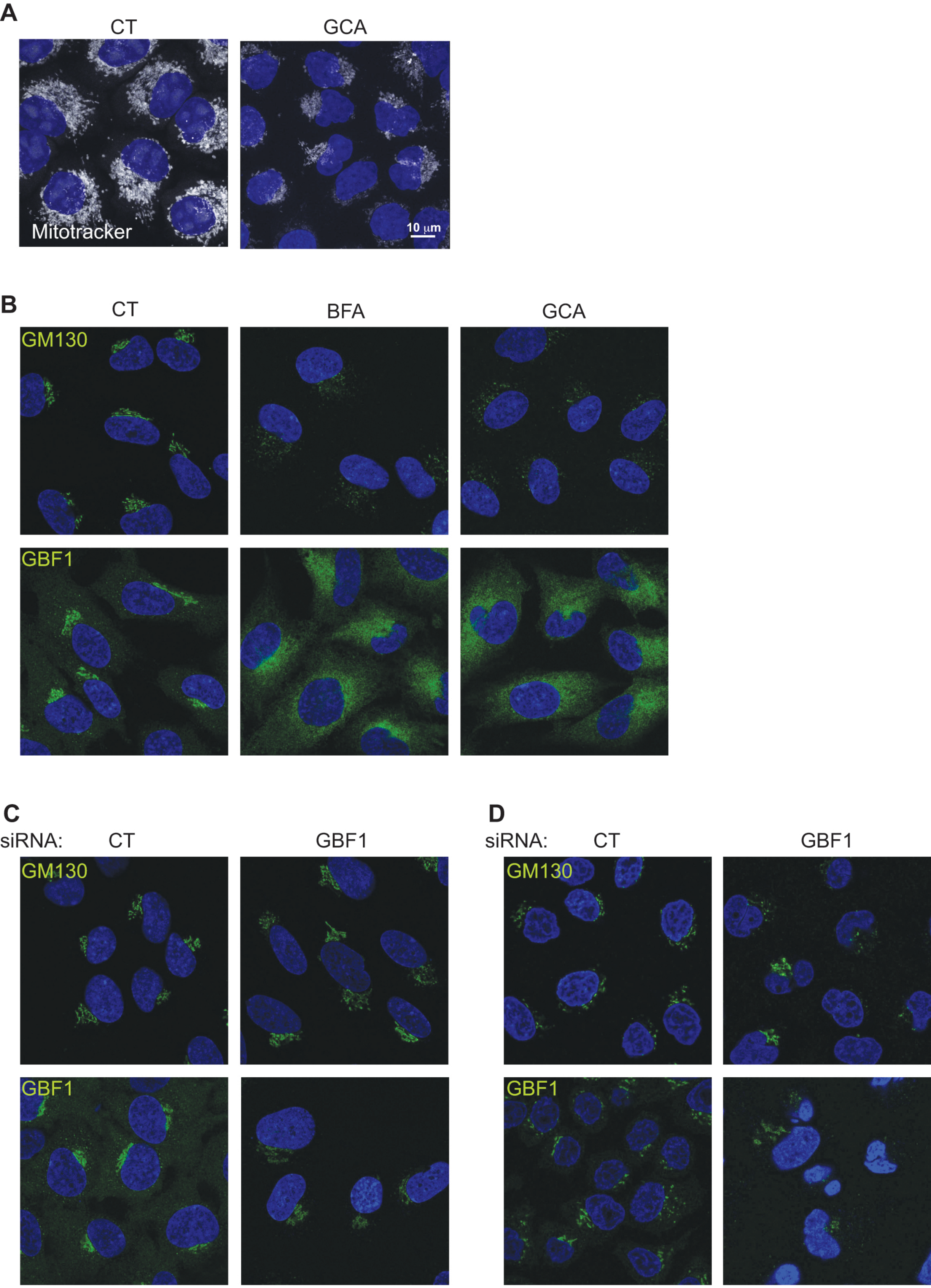
A
CT
GCA
Mitotracker
10 μm
B
CT
BFA
GCA
GM130
GBF1
C
siRNA:
CT
GBF1
GM130
GBF1
D
siRNA:
CT
GBF1
GM130
GBF1

Fig S2

Figure S2. Golgi and mitochondrial morphology in cells treated with GBF1 inhibiting drugs or siRNAs. The inhibition of GBF1 activity induces the condensation of the mitochondrial network in the perinuclear region and Golgi dispersion, whereas GBF1 silencing leaves Golgi remnants. (A) HeLa cells were incubated with either GCA (10 μM) or DMSO alone (CT) for 1h30, loaded with MitoTracker Orange for an additional 30 min and fixed. Cells were imaged using confocal microscopy. Images are maximum intensity Z-projections. Nuclei appear in blue (DAPI staining) and mitochondrial network in white. (B) RPE1 cells were incubated with either BFA (10 μg/ml), GCA (10 μM) or DMSO alone (CT) for 2h, fixed and stained for GM130, *cis*-Golgi marker, or GBF1 (green signal). Cells were imaged using confocal microscopy. Images are one Z-stack. Nuclei appear in blue (DAPI staining). RPE1 (C) or HeLa (D) cells transfected with GBF1, or irrelevant siRNA duplexes (CT), three days post-transfection, cells were fixed and stained for GM130 or GBF1 (green signal). Cells were imaged using confocal microscopy. Images are one Z-stack. Nuclei appear in blue (DAPI staining).

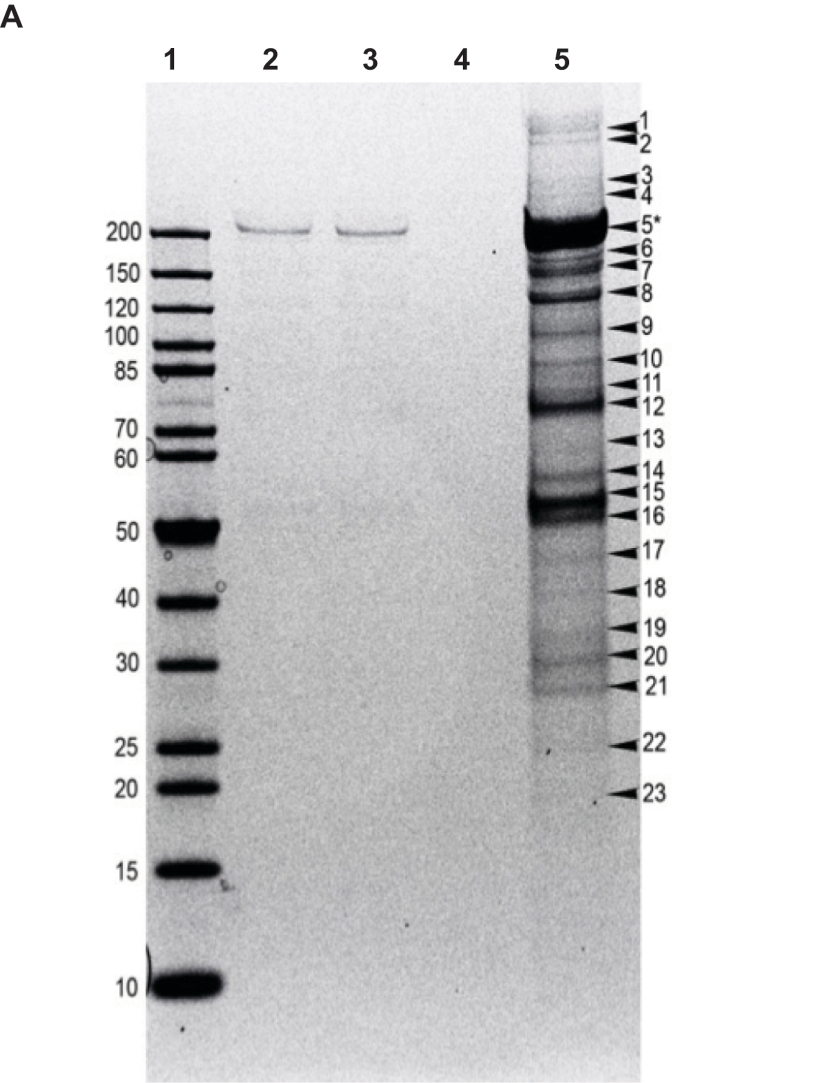
A
1
2
3
4
5
200
150
120
100
85
70
60
50
40
30
25
20
15
10
1
2
3
4
5*
6
7
8
9
10
11
12
13
14
15
16
17
18
19
20
21
22
23

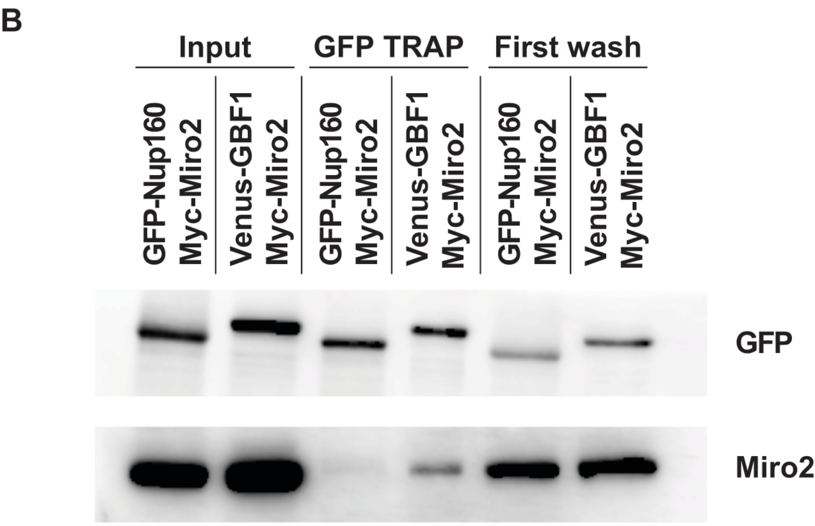
B
Input
GFP TRAP
First wash
GFP-Nup160
Myc-Miro2
Venus-GBF1
Myc-Miro2
GFP-Nup160
Myc-Miro2
Venus-GBF1
Myc-Miro2
GFP-Nup160
Myc-Miro2
Venus-GBF1
Myc-Miro2
GFP
Miro2

Fig S3

Figure S3. Co-immunoprecipitation of Miro with GBF1. (A) HEK293-FRT cells were transfected with pcDNA5/FRT/TO1-His-PC-TEV-GBF1. (His)6-PC-GBF1 was purified using a Protein C affinity resin, then eluted proteins were dialyzed and purified on a Ni^{2+}-sepharose resin. Proteins were separated on a preparative Coomasie gel, bands were cut out, then analyzed by Mass Spectrometry. GBF1 (24 peptides) and Miro2 (2 peptides) were identified in bands 5 and 12, respectively. Lane 1: MW markers, 2: protein eluate after incubation with Protein C beads, 3: eluate after the dialysis, 4: eluate depleted by Ni^{2+} beads, 5: Ni^{2+} beads boiled in SDS loading buffer. (B) RPE1 cells were cotransfected with GFP-tagged Nup160 or Venus-tagged GBF1 and Myc-Miro2. Immunoprecipitations were carried out with GFP-beads. Cells lysates (Input), immunoprecipitated proteins and the non-bound proteins after immunoprecipitation (First wash) were analyzed by Western blotting using anti-GFP and anti-Miro2 antibodies. The input and the first wash lanes represent 15% of lysate used in immunoprecipitation reactions.

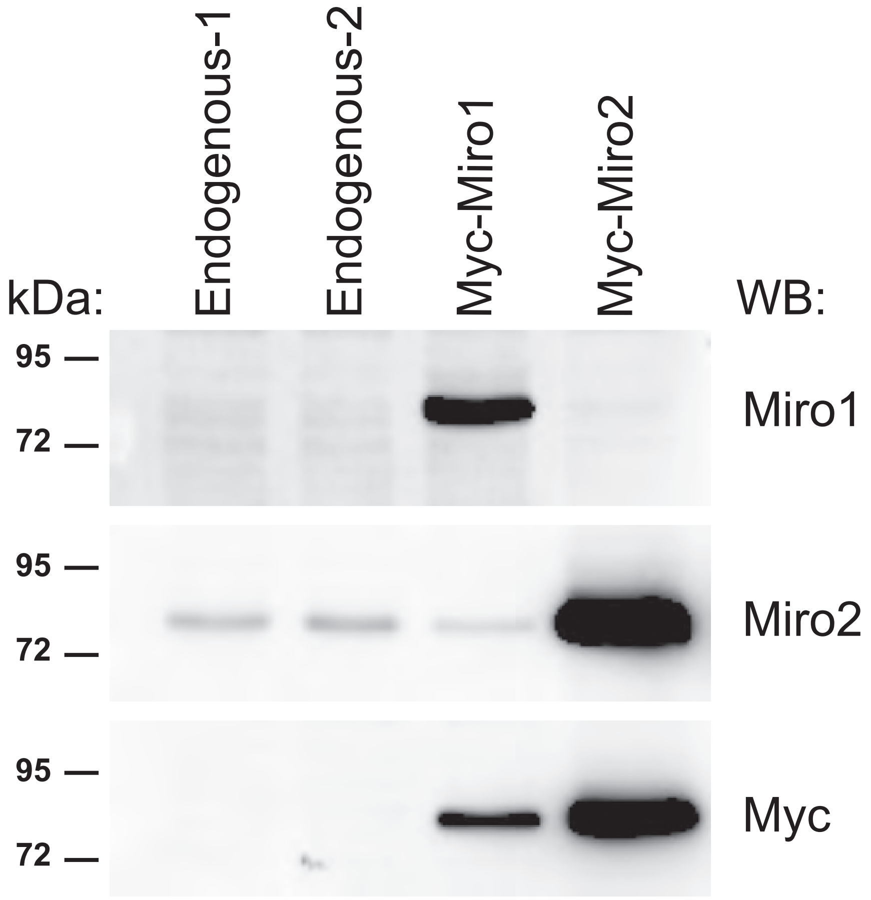

Figure S4. Miro2 is the predominant Miro isoform in RPE1 cells.
RPE1 cells were transfected with either Myc-Miro1 or Myc-Miro2. Endogenous and overexpressed Miro levels were analyzed by Western blotting using Miro1, Miro2 and Myc antibodies. 20 μg of protein were loaded per lane.

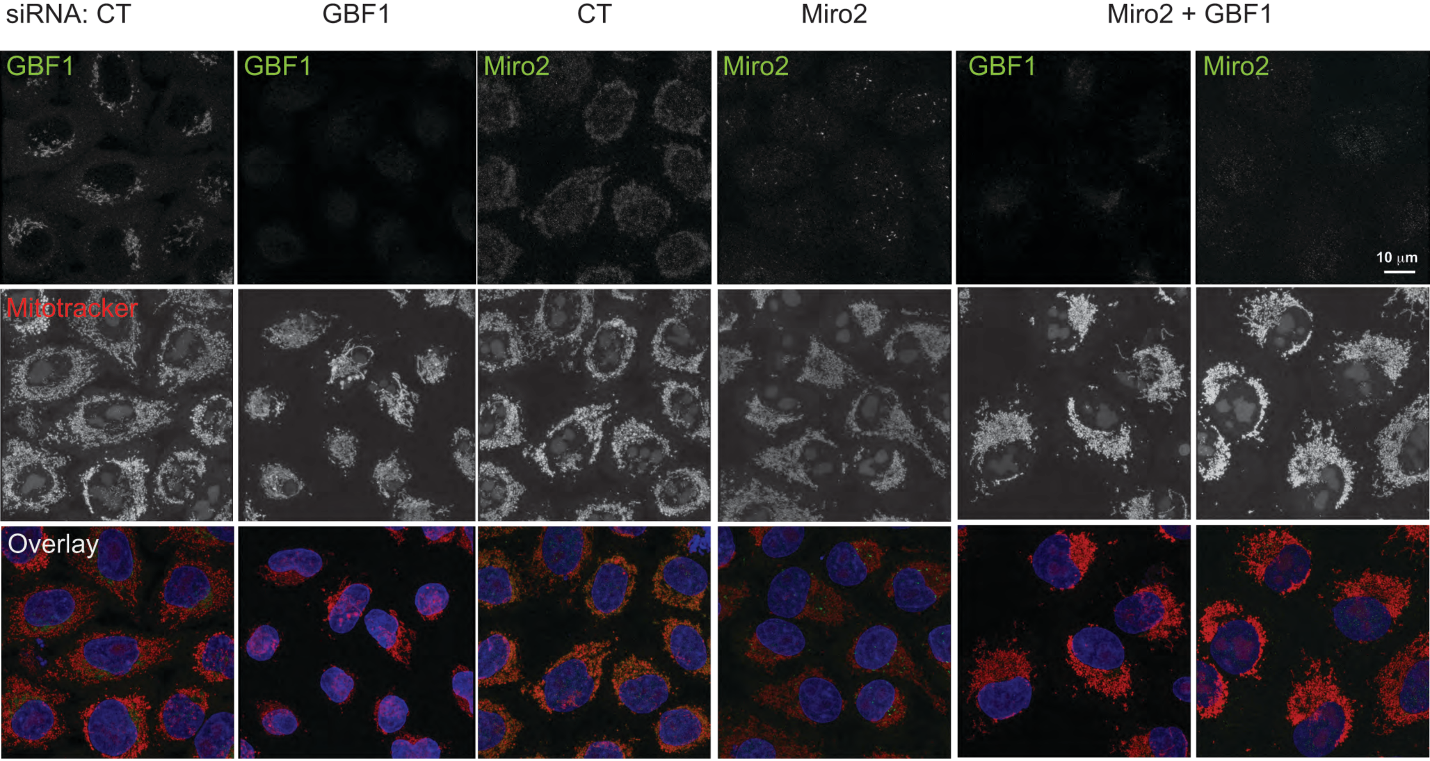

siRNA: CT
GBF1
CT
Miro2
Miro2 + GBF1
GBF1
GBF1
Miro2
Miro2
GBF1
Miro2
10 μm
Mitotracker
Overlay

Fig S5

Figure S5. Miro2 is required for mitochondrial condensation in HeLa cells knocked down for GBF1. HeLa cells were transfected with GBF1 or Miro2 alone, GBF1 and Miro2 or (CT) irrelevant siRNA duplexes. Three days post-transfection, cells were loaded with MitoTracker Orange, fixed and stained for GBF1 or Miro2 (green signal). Images are maximum intensity Z-projections.

Fig.4C

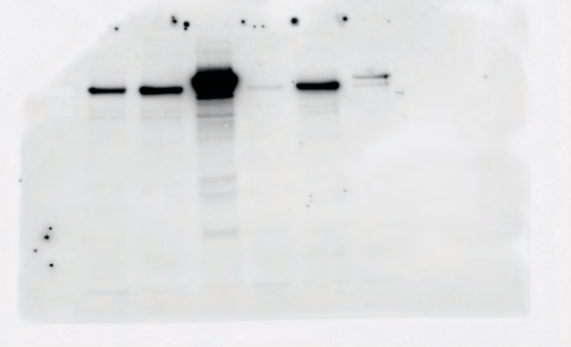

Fig.4C

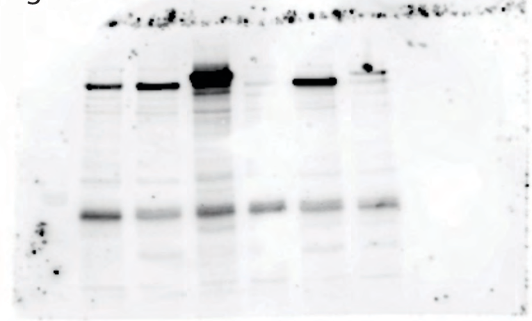

Fig.4C

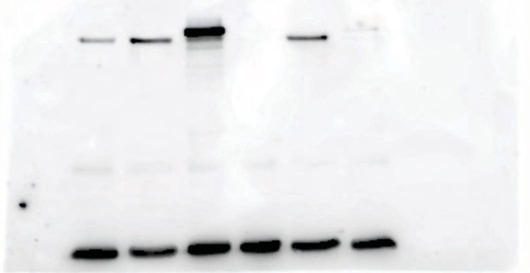

Fig.5A-B

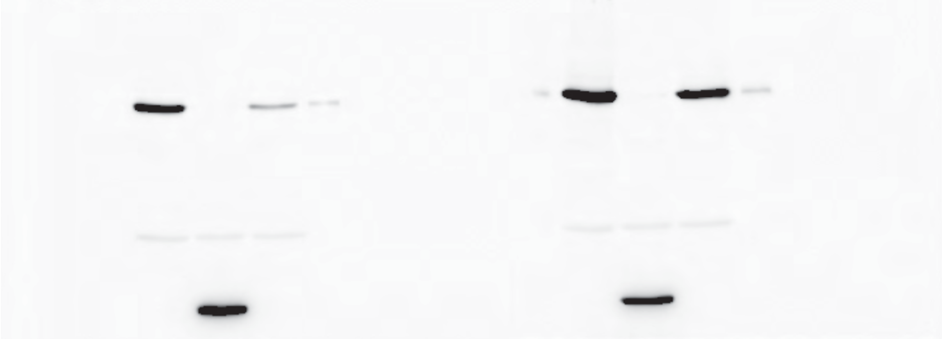

Fig.5A-B

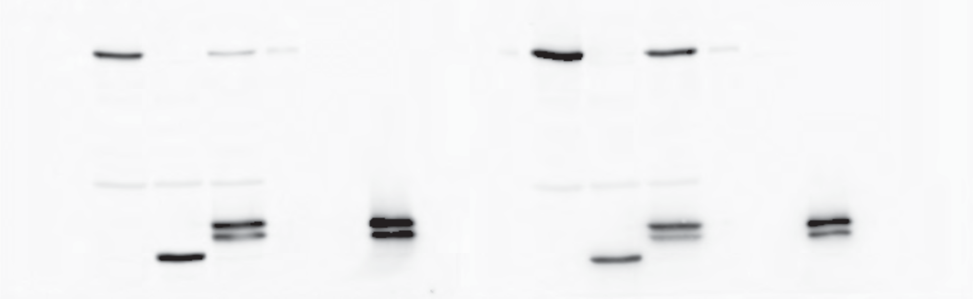

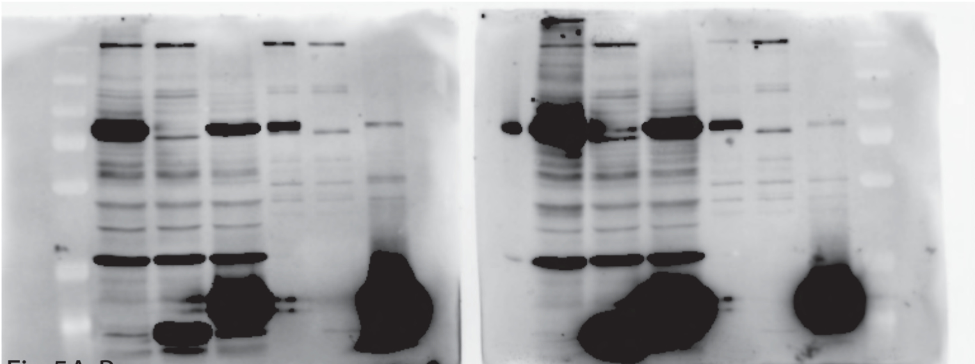

Fig.5A-B

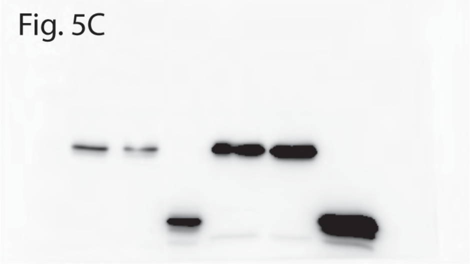
Fig. 5C

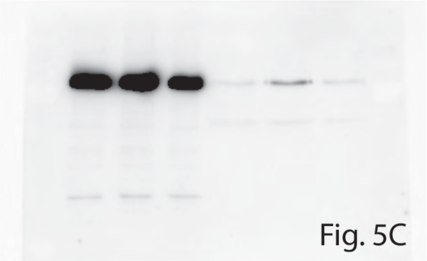
Fig. 5C

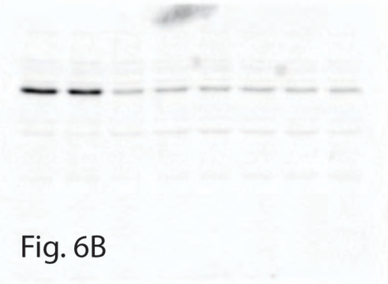
Fig. 6B

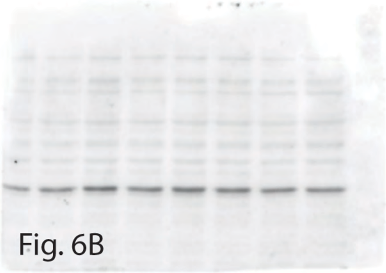
Fig. 6B

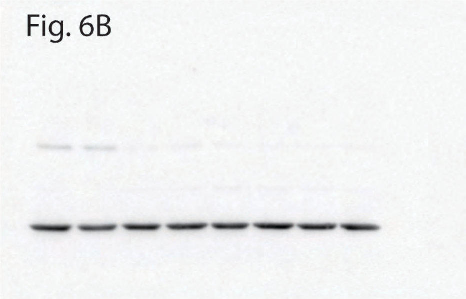
Fig. 6B

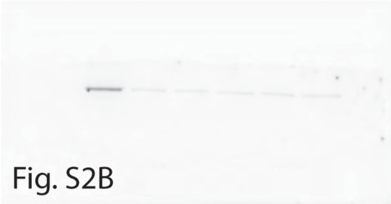
Fig. S2B

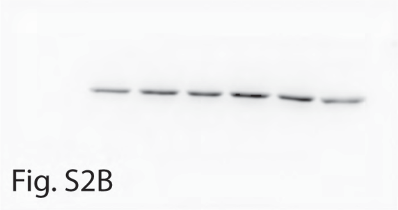
Fig. S2B

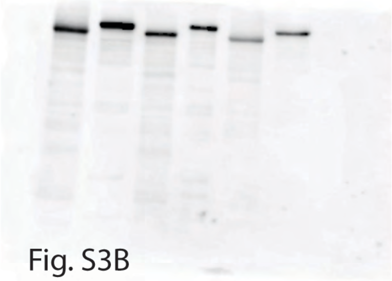
Fig. S3B

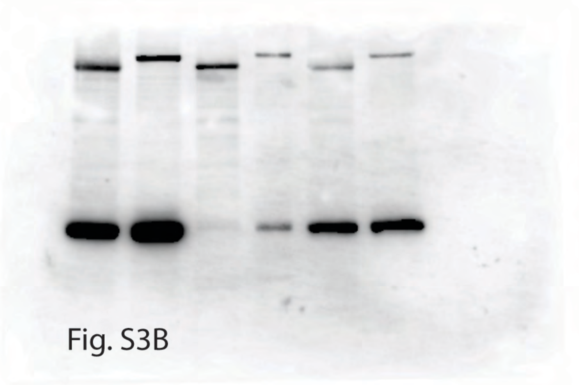
Fig. S3B

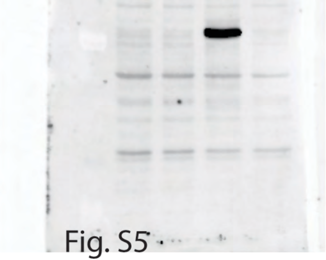
Fig. S5

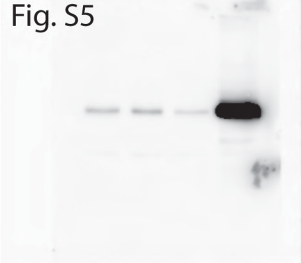
Fig. S5

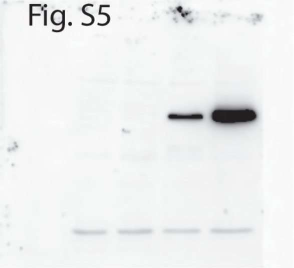
Fig. S5

Figure S6. Full-length blots. The full-length blots from which cropped portions were taken for display in the main and supplementary figures. The corresponding figure number is indicated on the blot.

Movie S1. Effect of BFA on mitochondrial dynamics. RPE1 cells were loaded with MitoTracker orange for 30 min at 37°C. At time 0, cells were treated with BFA (10 μg/ml), and images were recorded every 30 sec for 30 min.

Movie S2. Tracking of mitochondrial dynamics in control cells. RPE1 cells were loaded with MitoTracker orange for 30 min at 37°C. At time 0, DMSO was added to cells, and images were recorded every 30 sec for 30 min. Mitochondria were segmented in ImageJ prior to tracking.

Movie S3. Tracking of mitochondrial dynamics in GCA-treated cells. RPE1 cells were loaded with MitoTracker orange for 30 min at 37°C. At time 0, cells were treated with GCA (10 μM), and images were recorded every 30 sec for 30 min. Mitochondria were segmented in ImageJ prior to tracking.
